# The Missed Opportunity of Patient-Centered Medical Homes to Thrive in an Asian Context

**DOI:** 10.3390/ijerph18041817

**Published:** 2021-02-13

**Authors:** Shilpa Surendran, Chuan De Foo, Chen Hee Tam, Elaine Qiao Ying Ho, David Bruce Matchar, Josip Car, Gerald Choon Huat Koh

**Affiliations:** 1 Health Systems and Behavioral Sciences Domain, Saw Swee Hock School of Public Health, National University Singapore, 12 Science Drive 2, Singapore 117549, Singapore; ephfchu@nus.edu.sg (C.D.F.); ephtamc@nus.edu.sg (C.H.T.); hoqiaoying@gmail.com (E.Q.Y.H.); ephkohch@nus.edu.sg (G.C.H.K.); 2 Health Services and Systems Research, Duke—NUS Medical School, 8 College Road, Singapore 169857, Singapore; david.matchar@duke-nus.edu.sg; 3 Department of Medicine (General Internal Medicine), Duke University School of Medicine, 400 Morris Street 3rd Floor, Durham, NC 27701, USA; 4 Centre for Population Health Sciences, Lee Kong Chian School of Medicine, Nanyang Technological University, 11 Mandalay Road, Singapore 308232, Singapore; josip.car@ntu.edu.sg

**Keywords:** patient-centered medical home, primary care, general practitioners, policy, qualitative, family medicine clinic

## Abstract

In recent years, there is growing interest internationally to implement patient-centered medical homes (PCMHs), and Singapore is no exception. However, studies understanding the influence of contextual policy factors on the implementation of PCMHs are limited. We conducted 10 semi-structured in-depth interviews with general practitioners working in seven out of the nine PCMHs. Audio recordings were transcribed and analyzed by two study team members in NVivo 12 Software using grounded theory techniques. Power dynamics between the stakeholders and lack of shared decision-making among them in selecting the locale of the PCMH and formulating the practice fee and pharmacy structure were the key factors which negatively affected the implementation of PCMHs on a larger scale. Over time, lack of funding to hire dedicated staff to transfer patients and misalignment of various stakeholders’ interest to other right-siting programs also resulted in low number of patients with chronic conditions and revenue. Countries seeking to implement a successful PCMH may benefit from building trust and relationship between stakeholders, engaging in shared decision-making, ongoing cost-efficiency efforts, and formulating a clear delineation of responsibilities between stakeholders. For a healthcare delivery model to succeed in the primary care landscape, policies should be developed keeping mind the realities of primary care practice.

## 1. Introduction

Patient-centered medical home (PCMH) is a healthcare delivery model active in the United States for more than a decade. It is based on key attributes such as access to a primary care physician, patient-centeredness, comprehensive and coordinated care, accessible services, and a commitment to quality and safety [1]. PCMH is associated with not just positive health outcomes at the primary care level but also reduced healthcare utilization at the tertiary level [2,3]. Therefore, in 2011 Singapore’s Ministry of Health (MOH) introduced the Family Medicine Clinic (FMC) as its localized version of PCMH to enhance chronic disease management in the primary care level [4].

Primary care in Singapore is provided through a combination of an island-wide network of polyclinics operated by the public sector and clinics run by private general practitioners (GPs) who primarily serve as solo practices. There are currently 20 polyclinics and about 1700 GP clinics. Polyclinics are multi-doctor clinics offering a comprehensive range of subsidized healthcare services [5].

FMCs adopted the 2014 National Committee on Quality Assurance guidelines for PCMH [4]. FMCs were developed through public–private partnerships with like-minded private GPs and Regional Health Systems (RHS) [6]. An RHS is a geographical cluster of tertiary hospitals, primary, and community care partners [6]. This partnership lasts for three years, after which FMC will function as a private entity, i.e., privatization. Under this partnership, MOH provides seed funding for capital expenses (infrastructure) and operational expenses (rental of the floor space, utility bills, salaries of staff), while RHS supports clinical matters and governance to ensure the appropriate use of funds [6]. Most of the FMCs are strategically located, either in shopping malls or housing estates with connections to the public transport hub to improve accessibility [4]. First FMC started its operation in 2013, and the most recent one in 2017. Currently, eight FMCs are in operation following the closure of one in 2017 [7]. The healthcare delivery model currently dominating the private primary care sector in Singapore is the primary care network (PCN). A PCN organizes private GPs into groups to provide holistic care for patients with chronic conditions [8]. Table 1 illustrates the key similarities and differences between PCMH and PCN.

The literature on PCMH implementation has mostly focused on the barriers and challenges associated with the internal operations of the primary care practices, such as difficulties related to change management and transformation process, challenges in implementing electronic health records, inadequate performance measures, and insufficient funding, resources, and infrastructure within the practices [9].

However, according to organization theory, there are other factors, which affect the implementation of PCMH [10]. These factors, generally called contextual policy factors occur in a broader context and are but not limited to historical and political backgrounds, relationships and collaborations between various stakeholders, and value [10,11].

Only a handful of studies have explored the broader contextual policy factors affecting PCMHs’ implementation [12,13,14,15,16,17]. For example, studies [12,14,15,16,17] reported misalignment of practice payment models with PCMH strategies and lack of funding as factors impeding practices to transform to PCMHs. Additionally, studies [16,17] identified workforce policy issues and the need for developing shared vision as factors hindering and facilitating PCMH transformation, respectively. To add to this limited evidence base, this study’s objective is to identify the contextual healthcare policy factors that influence the implementation of FMCs on a larger scale in Singapore. We define contextual policy factors as factors that are external to the FMC, responsive to change, and that have a wider influence on the prioritization, formulation, and implementation of FMCs [11,16]. These factors include but are not limited to funding, relationship with stakeholders, decision-making process, and alignment of interest [11,16]. This study is important because, firstly, in an Asian context, PCMHs are implemented only in Singapore. Secondly, internationally, countries are moving towards implementing PCMH or PCMH-like initiatives to strengthen their primary care. Therefore, demonstrating the intricacies of implementing a PCMH in different environments will be beneficial for those working on such models of healthcare delivery in other countries [17].

## 2. Materials and Methods

### 2.1. Study Design and Setting

This qualitative study was conducted across seven FMCs in Singapore, including one that ceased operations in 2017. By including the FMC that ceased its operation, we were able to examine their perspective of contextual policy factors, which affected their operation. This study is reported using the Consolidated criteria for Reporting Qualitative research (COREQ) guidelines (Supplementary Material 1: COREQ Checklist) [18].

### 2.2. Participant Recruitment

We recruited a total of 10 participants; eight GPs and two nurse managers who are working or have worked in FMCs (Table 2). We approached 12 potential participants who were purposively selected based on their expertise, and their position within the FMC. We contacted them via email or telephone, stating the objectives of the study from April to November 2019. Two potential participants did not respond despite multiple emails.

### 2.3. Ethical Considerations

The Institutional Review Board of the National University of Singapore reviewed and approved this study (Reference code: S-19-005). We obtained written informed consent from the study participants.

### 2.4. Data Collection

We pilot tested the interview guide with two GPs who are working in FMCs and modified it before data collection (Supplementary Material 2: Interview guide). Some questions were rephrased. For example, the question can you describe the origins of FMC was rephrased to how did the current primary care schemes such as FMC come about? Additionally, we added a few more probes, such as the composition of FMC.

Semi-structured in-depth interviews were conducted in English at the participant’s workplace. Interviews were audio-recorded. One-on-one interviews were conducted over focus group discussions so that participants can share their views freely without feeling uncomfortable in the presence of their colleagues. Researcher (C.D.F.) conducted the interview while the researcher (S.S.) took detailed fieldnotes. Data collection and analysis were conducted in parallel until we reached data saturation by the 10th interview. Interviews lasted 60 to 80 minute in length on average. To ensure the study’s trustworthiness, firstly, the interviewer and the note taker acknowledged their position as a member of the research team with the participants to mitigate any pre-conceived bias arising due to their role in the research team [19]. Secondly, we contacted the participants in the final stage of data analysis to validate the researchers’ understanding of the data and verify the correct representation of participants’ views [20].

### 2.5. Data Analysis

An independent professional transcribed the interviews verbatim, and we checked the transcribed records for accuracy. Researchers (S.S., C.D.F.) applied techniques from grounded theory during the analysis. Before coding, we read and familiarized ourselves with the records. We individually conducted open-coding and line-by-line analysis of initial records to develop a coding frame using QSR NVivo 12 Software [21]. We used the initial coding frame to code a few randomly selected records. Discrepancies in coding were discussed and resolved among the research team members to improve analytic rigor. Through this iterative process, a final coding frame was developed and applied to the remaining records. Using a constant comparative method, we compared new data with existing codes. Thematic saturation was reached when no new codes, themes, or patterns emerged from the data. According to Guest et al. [22], in exploratory interviews, thematic saturation is reached after six to 12 interviews. Each quote includes FMC, followed by a number (FMC 1).

## 3. Results

Out of 10 participants, eight were males, and two were females. In total, we identified five themes and 57 codes. Among the five themes, the four policy themes that affected FMCs’ implementation are reported. These themes were identified based on the definition of the contextual policy factors, which is mentioned in the Introduction section of this paper. The four policy themes identified are (1) healthcare workforce (4 codes), (2) government funding and healthcare financing (10 codes), (3) power relations and decision-making (29 codes), and (4) alignment of interest and collaboration (11 codes). We did not report the fifth theme, i.e., information system (3 codes) since it does not fit in the definition of contextual policy factors. Table 3 summarizes the four policy themes, the issues under each theme, and the authors’ provided policy recommendations.

### 3.1. Themes

#### 3.1.1. Healthcare Workforce

Staffing pattern varied across FMCs, and participants described changes in staffing pattern before and after privatization. Before privatization, participants (2/10, 20%) mentioned that the RHS transferred case managers and care coordinators to FMCs to perform healthcare services and to manage patients with chronic conditions. During this time, many patients with chronic conditions were transferred to the FMCs. For example, 200 patients/month were transferred before privatization, and a few FMCs either had an increase in revenue or was able to break-even. Majority of the participants (8/10, 80%) reported that the RHS staff were not seconded to FMCs during the partnership years.

However, after privatization, many barriers significantly influenced FMCs implementation on a larger scale. For instance, RHS pulled back their hospital staff from the FMC firstly, due to workforce shortages across the healthcare continuum, and secondly due to no career advancement for their staff seconded to the FMCs. Lastly, RHS completely shifted its focus back to its primary aim of managing the hospitals. This drastically reduced the number of patients with chronic conditions (40 patients/month) transferred to FMCs. The verbatim was:

*“[..] a lot of the RHS say now I need to slowly pull my people back because primary care also very difficult to hire nurses, and you (FMC) don’t have very much career prospect for them. You also cannot expect the hospital always to be here because that is not their core, their core is just run the hospital right”* (FMC 5)

#### 3.1.2. Government Funding and Healthcare Financing

All participants (10/10, 100%) recognized MOH as FMCs’ funding body. One participant (10%) reported that some FMC GPs might not have considered money as a motivational factor in enhancing productivity. This participant reported that FMC GPs might not have felt an urgency to improve FMCs’ number of patients with chronic conditions resulting in FMCs sub-optimal performance even during the partnership years. On the contrary, another participant (1/10, 10%) attributed cessation of MOH funding to low transferred patient numbers after privatization. This is because FMCs hired salaried care coordinators, who were solely responsible for transferring patients with chronic conditions from Specialist Outpatient Clinics using MOH seed funding. Removing their headcounts after privatization and entrusting the responsibility of transferring patients to frontline hospital staff with a different job scope reduced the patient flow affecting FMCs implementation on a larger scale. Four out of 10 participants (40%) mentioned lack of funding for an integrated health information system as a reason specialist were not transferring many patients to FMCs. The verbatim was:

*“Principle of funding didn’t consider the motivating factors, too much of support […] you are killing the project […] get a bit layback, push and hunger is not there to make it work.”* (FMC 1)

Participants (6/10, 60%) reported that the implementation of the Community Health Assist Scheme (CHAS); a new healthcare financing reform has helped FMCs’ rollout nationwide. CHAS is a portable medical subsidy whereby citizens of Singapore can enjoy subsidized medical treatment at GP practices [26]. However, majority of the participants (8/10, 80%) mentioned that the CHAS quantum was insufficient to cover the cost of treatment at FMCs when the medical condition worsened because the healthcare services are unsubsidized at FMCs. Due to this, the number of patients with chronic conditions dropped at FMCs.

#### 3.1.3. Power Relations and Decision-Making

For this paper, we define power as the position of influence, be it asymmetric or evenly distributed among the stakeholders in the decision-making processes. Decision-making about FMC’s internal operations (practice fee and pharmacy structure), location, and floor size were predominantly made by RHS and hospital management according to more than half of the participants (6/10, 60%). Two FMCs were set up in a “quiet” location within a shopping complex, resulting in low footfall from poor visibility to the community that it served. Attempts to improve visibility were met with difficulties due to the lack of a marketing budget, hospitals rejecting the usage of their logo for publicity and push back by building owners from displaying posters around the shopping complex. After privatization, two FMCs downsized their clinic’s floor space since they could not sustain financially due to high rental costs and low patient numbers.

Practice fee at FMCs was either capped or fixed by their partner hospital according to some participants (3/10, 30%). Therefore, they (3/10, 30%) mentioned that FMCs were unable to translate the amount of time spent per consultation to revenue. Additionally, since patients with chronic conditions require extended consultation time, this translates to less revenue from the practice fee because only fewer patients can be seen overall. All FMCs increased their practice fee post-privatization and reported an increase in revenue. On the other hand, some participants (3/10, 30%) mentioned negotiating with their partner hospital to either have a separate practice fee for patients with acute consultations or to mark-up their practice fee for patients with chronic conditions. They mentioned having a break-even in revenue during the partnership years. All FMCs increased their revenue post-privatization and saw an increase in revenue.

Medications were either procured by a few FMCs from their partner hospitals or the pharmacy was owned by the partner hospitals according to half of the participants (5/10, 50%). This reduced the overall revenue generated by the FMCs from sale of medications because medications were sold at the subsidized rates listed at hospitals. Post-privatization, some FMCs explored innovative ways to increase their revenue. For example, some participants (3/10, 30%) reported that some FMCs either operated an additional private pharmacy solely for corporate and foreign patients or they completely discontinued operations with their partner hospital and adopted a private pharmacy altogether.

RHS and partner hospitals lacked experience managing a private healthcare entity, and they were unable to think from GPs’ perceptive. Therefore, one participant (1/10, 10%) mentioned that private GPs managing big private hospitals should be consulted and assigned the decision-making processes. Participants (2/10, 20%) reported that two FMCs had a deficit revenue of approximately 400,000 SGD (USD300,000) by the end of their partnership term and one FMC closed its operations. The verbatim was:

*“[…] because there isn’t many who have private sector experience in the government service and a GP […] know how a private system work […] what do not work […] a lot of these collaborations there is no private-public partnership in reality […] the private one is quiet and there’s no real private input. It (partnership) is actually for show. If MOH is interested in getting the health care up it, need to get some of these people who are in the private sector […] consult and even put them in-charge.”* (FMC 1)

Lack of autonomy for GPs to operate after office hours, and specialists lacking trust in GPs’ skills were also the reasons reported by participants (3/10, 30%), which hindered FMCs’ implementation on a larger scale.

#### 3.1.4. Alignment of Interest and Collaboration

For this paper, we define alignment as the agreement and mutual understanding between the stakeholders because of shared interests to achieve a state of optimal chronic disease management [23]. Alignment of GPs’ interest to manage patients with complex chronic diseases and the MOH’s focus to revamp primary care in response to the increasing chronic disease burden were reported as factors driving FMCs rollout nationwide.

Collaborations with healthcare and social service partners were reported as a natural outcome of this alignment. A reason for collaboration was to decrease health care costs by decreasing hospitalization rates according to many participants (6/10, 60%). During the partnership years, three FMCs received a significant number of patients with chronic conditions. These patients were transferred from their partner hospital. However, this patient flow diminished over time, and they received a few to no patients with chronic conditions despite their continued efforts to engage their partner hospitals. Government’s change in policy direction to build more polyclinics, lack of an incentive for RHS to transfer patients with chronic conditions to FMC and redistribution of patients with chronic conditions to other right-siting programs were the reasons reported by some participants (5/10, 50%) for the reduced patient flow. Declining transferred patient numbers and revenue were factors that made FMCs implementation challenging on a larger scale. The verbatim was:

*“[…] the interest in the FMCs have waned, and then some FMCs even close down because there’s not enough revenue and so on […] it’s just whether FMC is the flavor of the month or not. So, the PCN (primary care network) was the next thing that came out. […] Government has shifted focus […] to build more polyclinics […]”* (FMC 5)

## 4. Discussion

Our study demonstrates from GPs’ perspective that power dynamics between the partners negatively affected the implementation of FMCs on a larger scale. The evolution of FMCs varied over the initial three years. The majority of the FMCs had an initial increase in the number of patients with chronic conditions resulting in at least break-even to increase in revenue, while others had a deficit revenue or ended operations at the end of three years. However, misalignment of interest over time and lack of funding led to decreasing number of patients with chronic conditions that were transferred to FMCs. Post-privatization, all FMCs adjusted their practice fee, pharmacy structure, and/or downsized their clinic space for financial viability.

### Establishment and Development

FMCs were an idea borne out from the MOH, and FMCs were formed in line with MOH’s 2011 Primary care masterplan [27]. Due to this, MOH assigned the decision-making power to RHS, resulting in power imbalances between RHS and GP partners. MOH’s assignment of authority to RHS was also to ensure the appropriate use of MOH seed funds and to encourage competition between the RHS to draw out the best business model for providing patient care at the most cost effective and efficient manner [6,23]. In the case of FMCs, GPs were on the receiving end of policy implementation that is top-down rather than a bottom-up approach.

This is in contrast to PCMHs in the United States where the principles of PCMH were created and promoted by professional societies of the GP community and picked up by the policymakers and national healthcare thought leaders [16,28]. Most GP practices had the decision-making power in PCMH transformation even though they adopted guidelines under an organization’s direction [29]. Therefore, there has been a bottom-up approach to PCMH transformation. Firstly, this is a wise and practical approach because PCMHs are firmly rooted in the private primary care landscape. Secondly, in an American perspective, it will be highly unthinkable for the government to impose terms with the GP practices for traditional arrangements like pharmacy structure and practice fee. In the case of FMCs, inadequate consultation with GPs managing big private hospitals when implementing FMCs was viewed as a missed opportunity to gain their valuable insights according to our study findings. Since the RHS are government-linked and managed like not-for-profit organizations, their perspective and experience are primarily shaped by a knowledge of governmental processes [30]. Thus, the asymmetry in the distribution of decision-making power affected FMCs’ implementation on a larger scale. Shared decision-making might have averted this situation.

In the United States, for PCMH transformation, GP practices had to obtain buy-in from their staff members, empower and engage them in decision-making processes [31]. Therefore, building a strong relationship, and defining the roles and responsibilities of various stakeholders was imperative. Whereas in the case of FMCs, the top-down initiatives were primarily aimed at achieving performance objectives, instead of addressing the relational attributes between GPs and the RHS. Additionally, with multiple stakeholders’ involvement came the added complexity to the decision-making process [32]. Therefore, policies that encourage setting aside structured time in the form of meetings for reflection and sense-making should be enforced. This can foster trust and relationship with stakeholder groups, which is essential to increase one’s influence on decision-making ambits [24]. Building a robust relationship is also critical to develop new population level initiatives that can achieve the intended outcomes.

Funding was provided to FMCs to shield GPs from any financial catastrophe during the partnership years and provide an avenue for GPs who have either sold their practice to continue general practice or those without tangible assets to pursue general practice. On the other hand, in Australia, misalignment of funding with PCMH goals discourages GP practices from transforming to PCMH [17]. In simple words, no matter how attractive PCMH might appear as a model of care, GP practices will not transform to it if they must sacrifice their livelihood [16,17]. However, according to our study findings, some FMC GPs felt that funding possibly weakened GPs intrinsic motivation to make FMC’s operation more efficient. These findings are compatible with a large body of literature in psychology and behavioral economics, which cautions that extrinsic incentives could counter-intuitively result in a drop in performance by crowding out intrinsic motivations that are important for the desired behavior [33,34]. Additionally, offering extrinsic incentives could signal GPs that the funding bodies do not trust their intrinsic motivation [35]. This signal could decrease GPs’ intrinsic motivation to perform. Therefore, providing decremental levels of funding might have averted this situation. Nevertheless, our study findings also showed that extrinsic incentives do not necessarily reduce intrinsic motivation in all GPs. The differences observed within our study could be due to reputational concerns [36] and behavioral determinants such as self-efficacy and motives [37,38].

Fee-for-service remuneration in the Australian and Singaporean general practice is a disincentive for practices to implement a PCMH. However, their mechanism of action differs. In the Australian context, the fee-for-service funding does not fund GPs for PCMH strategies such as care coordination and non-face-to-face consultations [17]; whereas in Singapore, the fee of healthcare services is unsubsidized in GP practices. Therefore, patients with chronic conditions will need to pay higher fee for the healthcare services at GP practices compared to polyclinics. This pushes patients away from GP practices because of the high out-of-pocket payment [23]. Our study findings showed that CHAS quantum was insufficient for patients with chronic conditions to seek care at FMCs. Therefore, payment reforms are undoubtedly imperative. However, it is essential to learn which payment structure works best and in what environment. Therefore, establishing evidence based on how different payment structures influence healthcare delivery and vice-versa is necessary. This will better inform both payers and policymakers on which approach is best suited for them [39].

This study is not without its limitations. Firstly, we identified from GPs’ perspective several policy issues that affected the implementation of PCMHs, i.e., FMCs on a larger scale. Since we excluded the RHS and policymaker perspectives, we could not identify divergences from their perspectives and verify the actual interplay of factors. Secondly, the findings presented in this study may not showcase a comprehensive account of all the policy issues affecting the implementation of PCMHs. Due to the nature of qualitative research, caution should be taken when extending the findings to other countries. However, to address these limitations, firstly we explored on issues that are not explicit to any country; secondly, we interviewed participants from seven out of nine FMCs; and lastly, specific strategies were taken to ensure rigor, trustworthiness, and avoid interpretation bias as explained in the Methods section of this paper.

## 5. Conclusions

Implementation of PCMHs on a larger scale has been challenging in Singapore due to a host of factors. Our study identified how the contextual policy factors were understood and interpreted by the GPs to have affected PCMHs implementation. Power relations and lack of shared decision-making between the stakeholders on the traditional arrangements hindered PCMH’s ability to reach its full potential in managing patients with chronic conditions. Lack of funding and misalignment of various stakeholders’ interest over time were also the contributing factors. Countries seeking to implement a successful PCMH may benefit from building trust and relationship between stakeholders, engaging in shared decision-making, ongoing cost-efficiency efforts, and formulating a clear delineation of responsibilities between stakeholders. For a healthcare delivery model to succeed in the primary care landscape, policies should be developed keeping mind the realities of primary care practice [16].

## Figures and Tables

**Table 1:** Key similarities and differences between patient-centered medical home (PCMH) and primary care network (PCN).

	Patient-Centered Medical Home	Primary Care Network
Concept	One-stop care center providing investigative and diagnostic services for patients with chronic conditions	Group of GP clinics organize themselves into networks that support more holistic and team-based care for patients with chronic conditions
Set-up	Done in partnerships with RHS	Done either in partnerships with RHS or a group of GPs on their own
Location	One physical location	Various locations based on the clinics who join the network
Ancillary services	Integrated within the center	Mobile services or referred to nearby polyclinics or service providers
Patient load	Transferred patients from polyclinics and hospitals	Existing patients
Funding	MOH seed funding for capital and operational expenses	MOH funding for backend service and for GP leads for clinical and administrative services

GP—General Practitioner; RHS—Regional Health Systems; MOH—Ministry of Health.

**Table 2:** Sociodemographic characteristics of participants and FMC characteristics.

FMC	Age	Gender	Occupation	Specialty	Years of Experience in Primary Care	Type of Practice	Ownership	Location in Building	Number of Employees
FMC 1	52	M	GP	Family Medicine	17	PPP	Rent	3rd floor	6
	34	F	GP		2.5				
FMC 2	39	M	GP	Family Medicine	10	P	Rent	2nd floor	6
	55	F	NM	-	2.5				
FMC 3	49	M	GP	Family Medicine	20	P	Rent	1st floor	12
	37	M	GP		6				
FMC 4	-	M	GP	Family Medicine	21	CO	Rent	-	-
FMC 5	-	M	GP	Family Medicine	22	P	Rent	4th floor	20
FMC 6	39	M	GP	Family Medicine	10	PPP	Rent	3rd floor	16
FMC 7	62	F	NM	-	4	P	Rent	1st floor	18

FMC—Family Medicine Clinic; M—Male; F—Female; GP—General Practitioner; NM—Nurse Manager; PPP—Public-Private Partnership; P—Private; CO—Closed Operation.

**Table 3:** Themes, issues, and policy recommendations.

Theme	Issue	Policy Recommendation
Healthcare Workforce	RHS pulled back their staff from FMCs due to workforce shortages and lack of career advancement	Develop a central workforce pool at national/state level to deploy healthcare staff across care venues [23]
		PCMHs to invest in staff’s training and refresher programs. Opportunities for self-improvement will encourage staff retention
Government Funding and Healthcare Financing	Funding weakened intrinsic motivation	Policymakers to provide decremental levels of funding so that providers’ intrinsic motivation is preserved
	Lack of funding post-privatization resulted in removing staff’s headcount	Pilot innovative provider payment models to understand its effect on healthcare delivery and vice-versa
	Inadequate quantum of subsidies for patients	Policymakers to continuously review and refine healthcare financing structures to retain only the cost-efficient models
Power Relations and Decision-Making	Lack of shared decision-making on FMC’s implementation components	Stakeholders to set-aside structured time (meetings) to develop trust and relationship [24]
		Policymakers to clarify roles and responsibilities of stakeholders from the outset
Alignment of Interest and Collaboration	Misalignment of interest over time	Policies encouraging stakeholders to develop shared vision should be enforced
		Healthcare delivery models should be developed in collaboration with stakeholders from macro (political), meso (clinical and non-clinical professionals, managers) and micro (patients) levels for sustainability [25]

RHS—Regional Health Systems; FMC—Family Medicine Clinic; PCMH—Patient-Centered Medical Home.

## Data Availability

Records will not be shared to protect the anonymity of the participants. Readers who wish to gain access to the data can write to the corresponding author; data may be granted upon reasonable request.

## Supplementary Materials

COREQ checklist and Interview Guide are available online at https://www.mdpi.com/1660-4601/18/4/1817/s1.
