# Supplementary material for: The Missed Opportunity of Patient-Centered Medical Homes to Thrive in an Asian Context
Source: Int J Environ Res Public Health. 2021 Feb 13;18(4):1817. doi: 10.3390/ijerph18041817 (PMC7917999; doi:10.3390/ijerph18041817)
Supplement: Supplementary file 1 [file ijerph-18-01817-s001.zip › Supplementary material file 2 Interview Guide_revised.docx]

Supplementary Material 2: Interview Guide

| **Section I**: Introductory questions |
| --- |
| 1. To start, can you tell me more about yourself?  - Can you share more about your professional background? - Current role/roles and job scope? - Can you share about your educational background ? |
| **Section II**: Current Status of Primary Care in Singapore |
| 1. How does the functioning of the FMC facilitate primary care in Singapore? (prompts: ancillary services, funding, manpower etc.) 2. Can you describe the organization/s and capacities of the FMC? (prompt: composition) 3. How did the current primary care schemes such as FMC come about? 4. What do you think are the major challenges of the implementation of the FMC? Why? (prompts: funding, ancillary services, administrative support, team-based care etc.) 5. What do you think are the major disadvantages of the FMC? Why? |
| **Section III**: Future of Primary Care in Singapore |
| 1. Can you comment about the specific efforts to influence the future of FMC? 2. What factors have promoted or inhibited any of the efforts you mentioned? |
| Closing questions and remarks |
| 1. Before we end, do you have any final thoughts to share? 2. We have come to the end of the interview. Do you have any other questions? |

FMC – Family Medicine Clinic
